# Mapping of Quantitative Trait Loci Underlying Cold Tolerance in Rice Seedlings via High-Throughput Sequencing of Pooled Extremes

**DOI:** 10.1371/journal.pone.0068433

**Published:** 2013-07-30

**Authors:** Zemao Yang, Daiqing Huang, Weiqi Tang, Yan Zheng, Kangjing Liang, Adrian J. Cutler, Weiren Wu

**Affiliations:** 1 Department of Agronomy, College of Agriculture and Biotechnology, Zhejiang University, Hangzhou, Zhejiang, China; 2 National Research Council of Canada, Saskatoon, Saskatchewan, Canada; 3 Key Laboratory of Ministry of Education for Genetics, Breeding and Multiple Utilization of Crops, Fujian Agriculture and Forestry University, Fuzhou, Fujian, China; Pennsylvania State University, United States of America

## Abstract

Low temperature is a major limiting factor in rice growth and development. Mapping of quantitative trait loci (QTLs) controlling cold tolerance is important for rice breeding. Recent studies have suggested that bulked segregant analysis (BSA) combined with next-generation sequencing (NGS) can be an efficient and cost-effective way for QTL mapping. In this study, we employed NGS-assisted BSA to map QTLs conferring cold tolerance at the seedling stage in rice. By deep sequencing of a pair of large DNA pools acquired from a very large F_3_ population (10,800 individuals), we obtained ∼450,000 single nucleotide polymorphisms (SNPs) after strict screening. We employed two statistical methods for QTL analysis based on these SNPs, which yielded consistent results. Six QTLs were mapped on chromosomes 1, 2, 5, 8 and 10. The three most significant QTLs on chromosomes 1, 2 and 8 were validated by comparison with previous studies. Two QTLs on chromosomes 2 and 5 were also identified previously, but at the booting stage rather than the seedling stage, suggesting that some QTLs may function at different developmental stages, which would be useful for cold tolerance breeding in rice. Compared with previously reported QTL mapping studies for cold tolerance in rice based on the traditional approaches, the results of this study demonstrated the advantages of NGS-assisted BSA in both efficiency and statistical power.

## Introduction

Many traits of agronomic importance in crops, including those related to abiotic stress tolerance, are quantitatively inherited. The genomic regions containing genes controlling a given quantitative trait are known as quantitative trait loci (QTLs). QTL mapping is one of the most common approaches for the genetic study of quantitative traits, which provides the basis for map-based cloning of related genes and marker-assisted selection (MAS) in crop breeding. However, QTL mapping is usually carried out by genotyping a large number of individuals that are progeny of a biparental cross, which is labor-intensive, time-consuming and costly.

The strategy of bulked segregant analysis (BSA) proposed by Michelmore et al. [1] provides a simple and effective approach to rapidly search for markers linked to specific genes or QTLs affecting a trait of interest by genotyping only a pair of pooled DNA samples from two sets of individuals with distinct or opposite extreme phenotypes. Since 2000, high-throughput genotyping technologies based on microarray [2] and next generation sequencing (NGS) [3] have developed very quickly. Using these technologies, BSA can identify large numbers of markers linked to the target genes or QTLs. Based on these linked markers, the target genes or QTLs can be directly mapped by referring to reference genome sequences. Hence, with the availability of high-throughput genotyping technologies and with reference genome sequences from more and more species, BSA is becoming an increasingly useful approach for gene or QTL mapping.

Many studies on the methodology and application of the high-throughput genotyping-assisted BSA have been reported, but they have been mainly focused on qualitative traits [4]–[9] while those on quantitative traits are still very limited. Wolyn et al. [10] first proposed an approach named eXtreme Array Mapping (XAM), which combines microarray-based genotyping with BSA for QTL mapping. With a practical example in *Arabidopsis thaliana* and a simulation study, they demonstrated that the method is effective for mapping single major QTLs. Later studies in yeast (*Saccharomyces cerevisiae*) [11] and by simulation [12] also demonstrated the effectiveness of microarray-assisted BSA in mapping major QTLs. Ehrenreich et al. [13] first applied NGS to BSA for QTL mapping. By utilizing NGS-assisted BSA as well as microarray-assisted BSA, they mapped a number of QTLs for 17 chemical resistance traits in yeast (*S. cerevisiae*), showing that the methods are applicable to various quantitative traits with different levels of genetic complexity, ranging from simple ones influenced by a major locus to very complex traits affected by at least 20 loci. Magwene et al. [14] proposed a statistical framework for QTL mapping based on NGS-assisted BSA and applied it to analyzing colony morphology in yeast (*S. cerevisiae*) as a demonstration. Swinnen et al. [15] mapped three major QTLs and additional minor QTLs conferring ethanol tolerance in yeast (*S. cerevisiae*) using NGS-assisted BSA. The studies reported have been largely conducted in yeast (*S. cerevisiae*). Only until recently, a study was reported using NGS-assisted BSA for QTL mapping in rice, in which they mapped several QTLs underlying resistance to rice blast, grain amylose content and germination rate under low temperature, demonstrating the applicability of the approach to plants [16].

Low temperature is one of the main abiotic stresses in rice cultivation. Low temperature in early spring can lead to slower growth, discoloration, withering, or even seedling death. Therefore, improvement of Cold Tolerance at the Seedling Stage (CTSS) is important for stable rice production. CTSS in rice is a complex trait controlled by multiple genes [17], [18]. A number of QTLs underlying CTSS have been mapped in rice using traditional molecular marker technologies and QTL mapping methods [19]–[27], and two major QTLs have been fine mapped [25], [28], [29]. In this study, we chose rice CTSS as an example for investigating the feasibility and efficiency of NGS-assisted BSA for QTL mapping in plants. Since many QTLs for CTSS have been mapped in rice before, it is possible for us to evaluate the reliability of the QTLs mapped in this study by comparison. Compared with the study of Takagi et al. [16], our study was performed using a much larger segregating population and a pair of much bigger pools of extreme segregants as well as a much higher sequencing depth for the NGS-assisted BSA, hoping to exploit as far as possible the potential of the approach in the precision mapping of QTLs.

## Materials and Methods

### Plant materials

An F_3_ population was created for the experiment from a cross between a japonica rice variety Nipponbare, of which the whole genome sequence is publicly available, and an indica rice variety LPBG developed by Fujian Agriculture and Forestry University. Our preparative had indicated that Nipponbare is more tolerant to low temperature than LPBG.

### Seed sowing and planting

Pregerminated seeds of the mapping population were sown on clean sand contained in rectangular (35×25 cm^2^) plastic trays, with 225 seeds per tray (15 rows×15 seeds/row). Seedlings were grown at 25°C in a greenhouse. The sand was kept wet by watering daily with tap water and applying Yoshida nutrient solution [30] every 3 days after the seedlings had reached the two-leaf stage. One hundred seeds of each parental line were also sown to provide a reference.

### Identification of individuals with extreme phenotypes on cold tolerance

Seedlings with uniform growth performance at the three-leaf stage were treated with low temperature in a phytotron growth chamber under a cycle of 12-h light (15000 LX) and 12-h dark. The seedlings were initially exposed to 14°C for 2 h, followed by 12°C for 4 h and 10°C for 4 h. During this period, the seedlings that were the most sensitive to cold (i.e., became withered) were identified visually as the extremely sensitive (ES) individuals, and were transplanted to the normal growth condition for recovery. The remaining seedlings were kept in the chamber and the temperature continued to incrementally decrease from 9°C for 14 h, 8°C for 6 h and 7°C for 2 h. During this period, most of the seedlings died, but a small proportion of seedlings survived and appeared normal. The surviving seedlings were collected as the extremely tolerant (ET) individuals and transplanted to the normal growth condition for recovery. A total of 48 trays were screened in 6 batches, with 8 trays for each batch. In each batch, ∼70 ES and ET individuals were selected, each accounting for ∼4% of total seedlings tested.

### DNA isolation and sequencing

A segment of fresh leaf (∼0.02 g) was excised from each selected seedling. Leaf tissues from 430 ES and 385 ET seedlings were pooled separately to extract DNA using the CTAB method [31]. The two DNA pools were purified with the GenElute Plant Genomic DNA Miniprep Kit (Sigma-Aldrich, St. Louis, MO, USA), respectively. A genomic DNA library was prepared for each DNA pool using the Illumina TruSeq DNA Sample Preparation Kit (Illumina Inc., San Diego, CA, USA) according to the manufacturer's instructions. Each DNA library was sequenced using an Illumina Hiseq 2000 sequencing platform. All raw high-throughput sequencing data have been deposited in the SRA database with accession number SRP021494.

### Data processing and SNP identification

The raw DNA-seq reads were trimmed and filtered to remove low-quality sequences via a modified Mott trimming algorithm implemented by a custom Perl script that we wrote by referring to the CLC Genomics Workbench quality trimming tools. An error probability limit of 0.05 and an ambiguous nucleotide limit of 2 were applied and reads shorter than 25 bp were discarded. The preprocessed reads that passed the quality control were then aligned to the published rice (Nipponbare) reference genome (RGAP 7; http://rice.plantbiology.msu.edu/) using the program Bowtie 0.12.7 [32] with the following parameter settings: -n 2 -l 20 -e 100 –best –strata -a -m 1. Reads aligned to more than one position in the reference genome were filtered out. Based on the mapping files (SAM files) of both pools generated by Bowtie, SNP identification was performed using the Bayesian model implemented by the programs mpileup and bcftools of SAMTools [33] with the parameter of base PHRED quality filtering cutoff set to 20. To avoid the influence of segregation bias from the theoretical ratio (1∶1) between the two parental alleles and small sample size (sequencing depth) of individual SNPs on QTL analysis, the identified SNPs were further filtered according to the following requirements: 1) the overall Nipponbare allele frequency of a SNP in the whole pool (the two pools as a whole) was neither <20% nor >80%; 2) the total depth of a SNP in the whole pool was neither <100 nor >400; and 3) the depth of a SNP in each pool was not <40.

### QTL analysis

With the SNPs selected, QTL analysis was performed using the method proposed by Magwene et al. [14]. A sliding window with a fixed width of 1000 kb was used to calculate the value of statistic *G*′ at every SNP so as to identify the genomic regions that showed *G*′ peaks, which indicated the possibility of QTL existence. The *G*′ value at a SNP was calculated using the following formula under the condition that the SNP was located at the centre of a window:
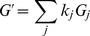
where *G_j_* and *k_j_* are the values of statistic *G* and weight *k* at the *j* ^th^ SNP, and the sum includes all SNPs within the window. The *G* value at a SNP was calculated using the following formula:
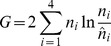
where *n*_1_ and *n*_2_, and *n*_3_ and *n*_4_, are the counts of the alleles from parent 1 (e.g. Nipponbare in this study), and parent 2 (LPBG), in the low trait value (ES) and the high trait value (ET) pools, respectively; and 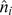 is the expected value for *n_i_* under the null hypothesis (*H*_0_: there are no QTLs linked to the SNP), e.g. 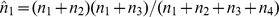. The *k* value at a SNP in a window was calculated using the following formula:
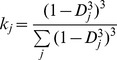
where *D_j_* is the standardized distance from the window centre to the *j* ^th^ SNP, of which the value varies from 0 (at the window centre) to 1 (at the window edge); and the sum again is for all the SNPs within the window.

The significance threshold of *G*′ was estimated using an emprical approach proposed by Magwene et al. [14] with a little modification. The approach is based on the assumptions that *G*′ asymptotically follows a log-normal distribution under the null hypothesis and the observed distribution of *G*′ is a mixture of the null distribution (in non-QTL regions) and several contaminating distributions (in QTL regions) [14]. The procedure is [14]: (1) Calculate *x* = ln(*G*′) for all *G*′ values. (2) Find Median(*x*). (3) Calculate *z* = Median(*x*)−*x* for all *x*≤Median(*x*). (4) Find Median(*z*). (5) Construct a trimmed data set of *G*′ values by discarding those *G*′ values for which *x*−Median(*x*)>5.2×Median(*z*). (6) Find Median(*G*′) and Mode(*G*′) in the trimmed data set and calculate the approximate estimates *μ* = ln[Median(*G*′)] and *σ* ^2^ = *μ*−ln[Mode(*G*′)] for the null distribution ln*N*(*μ*, *σ* ^2^). (7) Calculate the *p* values of all the *G*′ values using the estimated null distribution. (8) Sort the *p* values in the order of small to large, namely, *p*_1_<*p*_2_<…<*p_i_*<…<*p_n_*. (9) For a required false discovery rate (FDR) *q* ^*^, find the largest *i* (denoted as *k*) for which *p_i_*≤*q* ^*^×*i*/*n*. (10) Calculate the corresponding *G*′ value of *p_k_*, which is the approximate estimate of *G*′ threshold at the significance level of the required FDR.

The key point in the above approach is to estimate the null distribution parameters, *μ* and *σ* ^2^ of ln(*G*′), by treating the *G*′ values from the contaminating distributions as outliers, which need to be identified and removed. For this purpose, it is important to reduce the proportion of contaminating components in the mixture distribution as much as possible. Therefore, instead of using all the *G*′ values, we only chose those from the regions that were less likely to harbor QTLs for the estimation of the null distribution parameters. In addition, to reduce the influence of uneven distribution of *G*′ values in the genome and correlations between closely linked *G*′ values, we did not use the whole *G*′ values but randomly selected one *G*′ value every 200 kb from the putative non-QTL regions for estimating the null distribution parameters. This procedure was repeated 20 times, and their averages were taken as the estimates of the null distribution parameters. With the null distribution obtained, the probabilities of a subset of *G*′ values were estimated and the *p*-value threshold as well as the corresponding *G*′ threshold for the FDR of 0.05 was estimated. The *G*′ subset was obtained by randomly selecting one *G*′ value every 200 kb across the whole genome for the similar reasons mentioned above and a consideration that a space of 200 kb between testing points, which is approximately equivalent to 1 cM on average in rice, could provide sufficient resolution for QTL mapping, while the residual *G*′ values are redundant.

Apart from the above analysis, we also employed the method of Jensen-Shannon divergence to detect differential SNPs (regions with an imbalance between allelic contributions from the two parents) between the two pools with a Bonferroni FWER multiple test correction at an overall significance level of 0.05 implemented by the GeneSpring NGS (Agilent Technologies). The genomic regions with a high frequency of differential SNPs also indicated a high likelihood of containing QTLs and therefore could provide a validation for the result of the G′ analysis.

In addition, to identify which parent possesses the resistant allele of a putative QTL, the profile of Nipponbare allele frequency difference (NAFD) between the ET and ES pools was plotted using a 300-kb sliding window moving across the genome with a fixed step length of 10 kb. The Nipponbare allele frequency (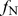) within a window in a pool was estimated using the following formula:
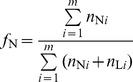
where *m* is the total number of SNPs within the window; and 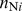 and 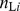 are the numbers of Nipponbare allele and LPBG allele of the *i* ^th^ SNP, respectively. For a putative QTL, the Nipponbare allele would act to increase the cold tolerance if NAFD>0, but to decrease the cold tolerance if NAFD<0.

## Results

In total, 10,800 F_3_ seedlings from the cross between rice varieties Nipponbare and LPBG were tested for cold tolerance, from which 430 ES and 385 ET individuals were selected and a pair of DNA pools was obtained. Through high-throughput sequencing of the two DNA pools, a total of ∼800 M 101-bp pair-end reads were obtained, with approximately 360 M and 440 M from the ES and ET pools, respectively (Table 1). After trimming and filtering, over 99% of the reads were selected in both pools, with the average length reduced to ∼98 bp. About 70% of the selected reads were mapped to unique positions in the reference (Nipponbare) genome (Table 1). These uniquely mapped reads covered ∼92% of the genome in both pools, with an average depth of ∼70 and 89 in the ES and ET pools, respectively, or ∼158 altogether (Table 2).

**Table 1:** Statistics of sequencing results.

DNA pool	Number of raw reads	Trimmed and filtered reads		Uniquely mapped reads	
		Number	%	Number	%
ES	356,193,956	353,856,120	99.34	244,801,282	69.18
ET	442,404,686	439,177,251	99.27	309,826,517	70.55
Total	798,598,642	793,033,371	99.30	554,627,799	69.94

**Table 2:** Coverage of the rice genome by the uniquely mapped reads.

DNA pool	Coverage length (bp)	Coverage rate (%)	Total length of reads (bp)	Coverage depth
ES	342,446,650	91.75	24,152,244,325	70.53
ET	340,022,302	91.10	30,351,271,370	89.26
Total	344,387,370	92.27	54,503,515,695	158.26

Note: The size of the reference genome is 373,245,519 bp.

**Figure 1:**
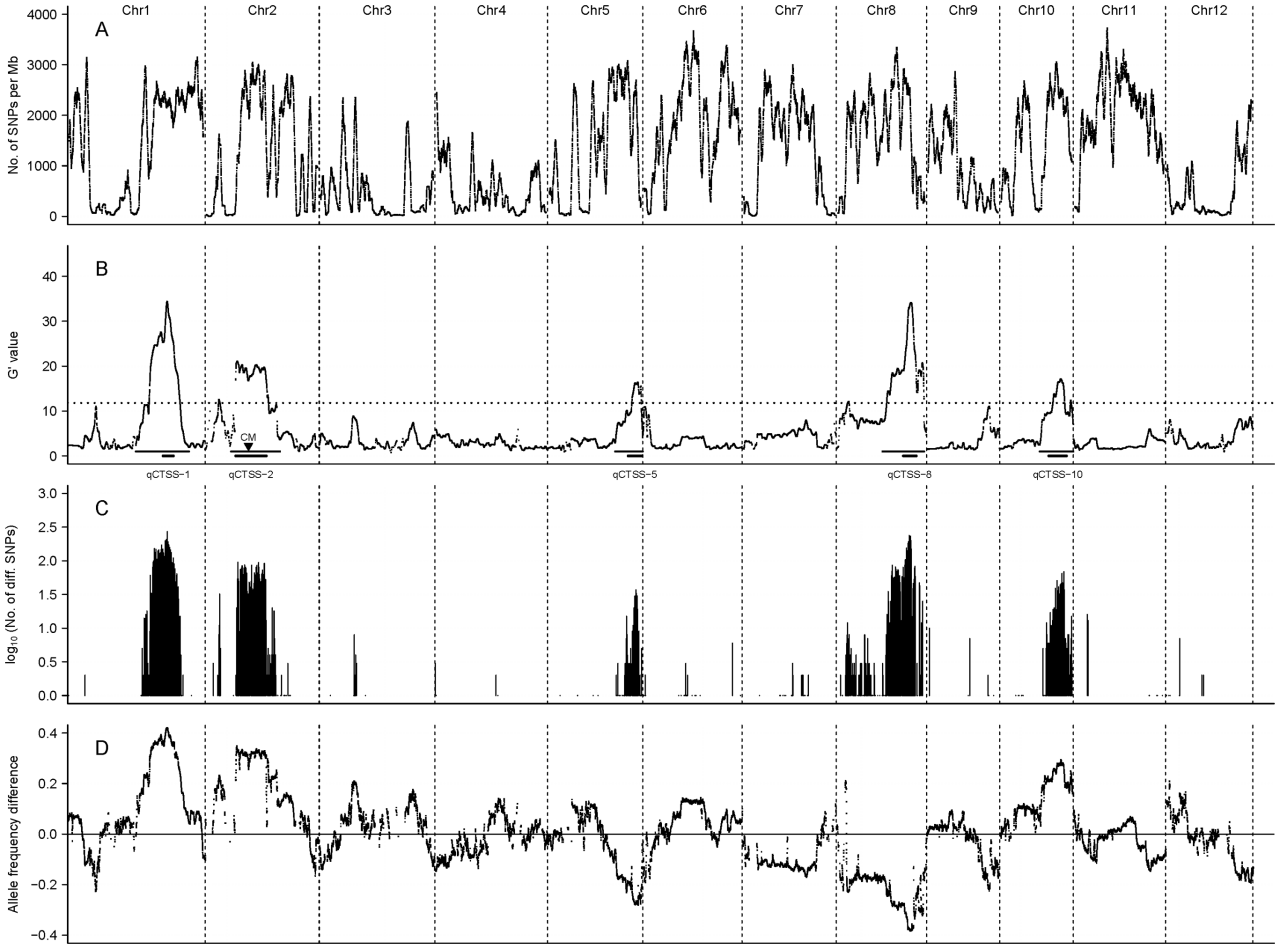
Statistical difference between the two pools along the genome revealed by three methods. A: SNP distribution in the genome. B: *G*′ value profile. The horizontal dotted line shows the significance threshold for FDR≤0.05. The upper longer and lower shorter horizontal bars under each major *G*′ peak indicate the ranges of the full and the most probable intervals of a putative QTL, respectively. The downward black arrowhead marked as CM within the interval of *qCTSS-2* indicates the position of centromere on chromosome 2. C: Distribution of differential SNPs in the genome. D: Profile of Nipponbare allele frequency difference.

Based on the uniquely mapped reads, a total of 456,777 SNPs met the chosen requirements for QTL analysis (see Materials and methods). These SNPs showed an approximately symmetric unimodal distribution of the Nipponbare allele frequency in the whole pool with an average of ∼45%, which was close to the expected value 50%, suggesting that the genetic segregation of these SNPs was approximately normal. However, the distribution of these SNPs in the genome was not even. The SNP density (shown as the number of SNPs per Mb or within the sliding window) varied greatly within the genome (Figure 1A).

**Figure 2:**
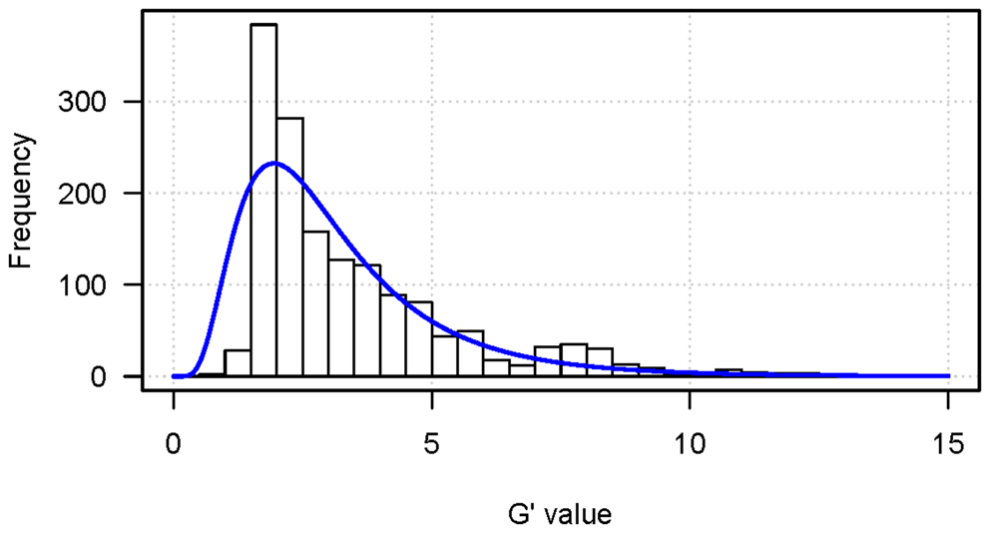
Frequency histogram and estimated null distribution of *G*′ values. The *G*′ values were sampled by randomly selecting one every 200 kb from the whole genome excluding the five major *G*′ peak regions.

Calculation of *G*′ values at these SNPs showed that there were five large major *G*′ peaks located on chromosomes 1, 2, 5, 8 and 10, with the peaks on chromosomes 1 and 8 being the highest (Figure 1B), suggesting that these five major *G*′ peaks were likely QTL regions. Hence, we excluded them and only selected *G*′ values from other regions to estimate the null distribution of *G*′. The obtained null distribution was ln*N*(0.9945, 0.3315) (Figure 2), according to which the significance threshold of *G*′ for the FDR of 0.05 was estimated to be 11.81. The five major *G*′ peaks with putative QTLs all greatly exceeded the threshold (Figure 1B), indicating that these five major *G*′ peak regions in high likelihood contain QTLs.

Jensen-Shannon divergence analysis indicated that the differential SNPs between the two pools were basically distributed in the five major *G*′ peak regions, showing profiles consistent with the corresponding *G*′ peaks (Figure 1C). These results further support the assertion that the five major *G*′ peak regions contain QTLs. We named these QTLs as *qCTSS-1* (abbreviation of “quantitative trait locus for CTSS on chromosome 1”; similar for others), *qCTSS-2*, *qCTSS-5*, *qCTSS-8* and *qCTSS-10*, respectively.

Apart from the five major *G*′ peaks, there were several small (minor) *G*′ peaks scattered throughout the genome (Figure 1B). Four of them were at the margin of significance, with two (towards the left end of chromosomes 2 and 8) slightly exceeding and two (one at the left end of chromosome 1, the other at the right end of chromosome 9) nearly reaching the threshold; while all others were apparently under the threshold (Figure 1B). For reliability, we only took the major *G*′ peaks as QTLs.

The full intervals of the QTLs (or the regions covered by the major *G*′ peaks) were all very wide, ranging from 8.68 Mb for *qCTSS-5* to 15.36 Mb for *qCTSS-2* (Figure 1B; Table 3). However, it can be seen that each of the major *G*′ peaks exhibited a clear “shoulder”, which divided the peak into two distinct parts, the “head” part over the “shoulder” and the “body” part under the “shoulder” (Figure 1B). Obviously, each QTL should be most probably located within the region covered by the “head” part of the corresponding *G*′ peak. Hence, we took the “head”-covered regions as the most probable intervals of the QTLs, which were all much narrower than the full intervals except for *qCTSS-2* (Figure 1B; Table 3).

**Table 3:** QTLs conferring cold tolerance at seedling stage in rice mapped in this study.

QTL	Position interval (Mb)		Summit	Summit	Source of
	Full	Most probable	G′ value	NAFD	resistant allele
*qCTSS-1*	21.45–38.22	30.09–33.28	34.38	0.41	Nipponbare
*qCTSS-2*	8.21–23.57	9.63–19.27	21.11	0.33	Nipponbare
*qCTSS-2a*	8.21–13.61	9.63–13.61	21.11	0.33	Nipponbare
*qCTSS-2b*	13.61–23.57	13.61–19.27	20.27	0.32	Nipponbare
*qCTSS-5*	21.28–29.96	25.40–29.63	16.45	−0.28	LPBG
*qCTSS-8*	14.57–27.68	21.14–25.17	34.18	−0.35	LPBG
*qCTSS-10*	12.64–23.19	15.48–21.06	17.14	0.30	Nipponbare

The profile of NAFD between the ET and ES pools (Figure 1D) was consistent with the *G*′ profile (Figure 1B). The summit NAFD value of a QTL can reflect the size of the effect and the direction of action of the Nipponbare allele of the QTL. It can be seen that *qCTSS-1* showed the largest effect, followed by *qCTSS-8*, *qCTSS-2*, *qCTSS-10* and *qCTSS-5*; and the resistant alleles of *qCTSS-1*, *qCTSS-2* and *qCTSS-10* were from Nipponbare, while those of *qCTSS-5* and *qCTSS-8* were from LPBG (Figure 1D; Table 3). This is consistent with the fact that Nipponbare is more tolerant than LPBG to low temperature stress (see Materials and Methods).

It is noticeable that the major *G*′ peak on chromosome 2 displayed a wide fluctuating top without a clear peak and appeared to be a mixture of two adjacent component peaks divided by a shallow valley (Figure 1B). By referring to NCBI mapview (http://www.ncbi.nlm.nih.gov/ mapview/), we found that the lowest point of the valley (at ∼13.61 Mb) happened to fall inside the centromere region (ranging approximately 13.57–13.75 Mb), where no genes were annotated. Therefore, the putative QTL in this region could only be located at either side of the centromere. However, if this was the case, it can be expected that the *G*′ profile in this region would exhibit a clear sharp peak and there would be no valley at the centromere. Thus, in light of the result, we suspect that there might be two QTLs harbored in this region, with one on each side of the centromere (named as *qCTSS-2a* and *qCTSS-2b*, respectively; Table 3). These two QTLs must be linked in coupling phase, with both resistant alleles coming from Nipponbare. The two component peaks that represented the two QTLs could not be clearly separated from each other, probably because of the generally low recombination rate in the centromere region.

Comparing Figure 1A and B, we see that there is no obvious correlation between *G*′ value and SNP density. However, it can be found that the major *G*′ peak regions all have high (or at least not very low) SNP density, and there were few large *G*′ values found in the regions with very low SNP density. This implies that *G*′ values tend to be underestimated within regions of very low SNP density. We have noticed that the SNP density was very low in the region between the minor and the major *G*′ peaks on chromosome 2. This means that the *G*′ values in this region might be underestimated. If this was true, the minor *G*′ peak might be an artefact and actually be a part of the major *G*′ peak.

## Discussion

Traditional QTL mapping methods require that every individual in a mapping population is genotyped and phenotyped. Genotyping with traditional molecular marker technologies is laborious and time-consuming and therefore has been a bottleneck for QTL mapping studies. In recent years, NGS technologies have been utilized to genotype mapping populations [34], [35]. This greatly increases the efficiency of genotyping and enables the construction of a genetic map with very high marker density. Hence, it would be very useful for QTL mapping. However, genome resequencing for hundreds of individuals is expensive and laborious. This limits the size of the population that can be used for QTL mapping. In contrast, NGS-assisted BSA only involves a pair of pooled DNA samples. Therefore, it is much cheaper and less laborious and has no limitation on population size for genotyping work.

In some cases, phenotyping can also be a bottleneck for QTL mapping studies, especially for the traits that are difficult to quantify. The target trait of this study is an example. CTSS is a complex trait, which is difficult to measure precisely and quantitatively, and is usually measured with various artificial scale systems such as the 1–9 scale system [19], [26] and the 0–4 scale system [24] or some related indices such as seedling survival percentage [20], [22], seedling mortality [23] or cold response index calculated based on seedling vigor traits [21]. All of these indices of CTSS can only be measured based on lines. Therefore, rice QTL mapping studies for CTSS so far were all conducted based on populations of recombinant inbred lines, doubled haploid lines and F_2∶3_ lines [19]–[26]. Compared with the traditional QTL mapping methods, NGS-assisted BSA has the very desirable feature that it does not require the precise trait value of each individual (or line) but only requires identification of the individuals (or lines) that exhibit opposite extreme phenotypes. This is not difficult in general because opposite extreme individuals (or lines) can usually be distinguished easily. In addition, BSA is more insensitive to the occasional phenotyping mistake [4]. This means that the requirement for phenotyping in NGS-assisted BSA is less stringent than in the traditional QTL mapping methods. Hence, QTL mapping by NGS-assisted BSA can be carried out based on a population of individuals rather than lines, even for such trait as CTSS, as demonstrated in this study.

Taken together, it is clear that NGS-assisted BSA makes both genotyping and phenotyping much easier and cheaper and thus greatly simplifies and accelerates QTL mapping. In addition, NGS-assisted BSA allows using a very large population, which increases the statistical power [13].

In this study, using a very large F_3_ population, we mapped 6 QTLs conferring CTSS in rice by NGS-assisted BSA (Table 3). The number of QTLs mapped by traditional QTL mapping methods is generally smaller, varying between 0 and 6, with an average of 3.0, according to previous studies of QTL mapping for CTSS in rice [17]–[24]. These results suggest that NGS-assisted BSA could be equally or more powerful than the traditional QTL mapping.

Among the 6 QTLs mapped in this study, the three most significant ones (*qCTSS-1*, *qCTSS-2b* and *qCTSS-8*) have been detected before (Table 4). For comparison, we identified the physical positions of the flanking markers of reported QTLs by referring to the Gramene database (http://www.gramene.org/) or by searching the rice genome with the markers' primer sequences using the BLAST program. By comparing Tables 3 and 4, it can be seen that the marker intervals of *qCTS1*, *qCTS-2* and *qCTS8-1* are all contained in the full intervals and largely overlapped with the most probable intervals of *qCTSS-1*, *qCTSS-2b* and *qCTSS-8*, respectively. These results indicate the reliability of QTL mapping based on NGS-assisted BSA.

**Table 4:** Common QTL regions conferring cold tolerance in rice identified in the present study and previous studies.

QTL	Acting stage	Marker interval (Mb)	Reference
*qCTSS-1*			
*qCTS1*	Seedling	RM297-RM319 (32.10–33.68)	[19]
*qCTSS-2a*			
*qCTB2*	Booting	RM324-RM301 (11.39–12.22)	[37]
*qCTSS-2b*			
*qCTS-2*	Seedling	RM561-RM341 (18.77–19.34)	[22]
*qCTSS-5*			
*qCTB5*	Booting	RM26-RM334 (27.40–28.55)	[37]
*qCTB-5-1*	Booting	RM7452-RM7271 (26.99–27.03)	[36]
*qCTB-5-2*	Booting	RM19106-RM31 (27.89–28.61)	[36]
*qCTSS-8*			
*qCTS8-1*	Seedling	RM284-RM230 (21.14–25.84)	[19]

The other three QTLs identified in this study are novel for the CTSS trait in rice. However, QTLs controlling cold tolerance at the booting stage have been reported in the regions of *qCTSS-2a* and *qCTSS-5* (Table 4). It can be seen that the position intervals of these reported QTLs (Table 4) coincide with those of *qCTSS-2a* and *qCTSS-5* (Table 3), implying that they might be the same QTLs. This result further verifies the reliability of NGS-assisted BSA in QTL mapping. In addition, it also suggests that some QTLs controlling cold tolerance may function at different developmental stages in rice. A similar result has been reported before [34]. These QTLs would be particularly useful for cold tolerance breeding in rice.

Another merit of using NGS-assisted BSA for QTL mapping is that the genomic sequence data obtained by NGS allows identification of the allelic variation (polymorphisms) between the parents, which will facilitate subsequent fine mapping and positional cloning of the QTLs. With the large number of identified polymorphisms, it will be easy to develop appropriate markers for the marker-assisted breeding of isogenic lines, which are generally required for fine mapping of QTLs. In addition, the identified sequence variations in genes combined with the information such as gene annotation and gene expression from other sources will be helpful for the identification of candidate genes of the QTLs. In this study, we identified all the genes that show amino acid variations between the parental varieties and significant responses to low temperature stress within the most probable intervals of the mapped QTLs (Tables S1, S2, S3, S4, S5, S6 in File SI), which could serve as the preferred list of candidate genes for these QTLs in further studies.

## Supporting Information

File S1 Tables S1, S2, S3, S4, S5, S6.
(XLS)
